# Brain-Derived Neurotrophic Factor, Neutrophils and Cysteinyl Leukotriene Receptor 1 as Potential Prognostic Biomarkers for Patients with Colon Cancer

**DOI:** 10.3390/cancers13215520

**Published:** 2021-11-03

**Authors:** Syrina F. Mehrabi, Souvik Ghatak, Lubna M. Mehdawi, Geriolda Topi, Shakti Ranjan Satapathy, Anita Sjölander

**Affiliations:** Cell and Experimental Pathology, Department of Translational Medicine, Lund University, 205 02 Malmö, Sweden; syrina.mehrabi@med.lu.se (S.F.M.); souvik.ghatak@med.lu.se (S.G.); lubna.mehdawi@med.lu.se (L.M.M.); geriolda.topi@med.lu.se (G.T.)

**Keywords:** BDNF, LTD_4_, CysLT_1_, montelukast, neutrophils, colon cancer

## Abstract

**Simple Summary:**

Colorectal cancer (CRC) is one of the most common type of cancer and the third leading cause of cancer-related death. CRC is associated with inflammatory bowel disease. We have earlier shown that high levels of the inflammatory receptor CysLT_1_ goes with poor prognosis for CRC patients. In this study, we found that high levels of neutrophils (CD66b) and brain-derived neurotropic factor (BDNF) goes with poor prognosis for colon cancer patient. We discovered a strong positive correlation between CysLT_1_, CD66b and BDNF. Our data support that these three proteins can be used as a combined biomarker for CC patients.

**Abstract:**

The tumor microenvironment has been recognized as a complex network in which immune cells play an important role in cancer progression. We found significantly higher CD66b neutrophil expression in tumor tissue than in matched normal mucosa in the Malmö colon cancer (CC) cohort and poorer survival of stage I-III patients with high CD66b expression. Additionally, mice lacking CysLT_1_R expression (*cysltr1^−/−^*) produce less brain-derived neurotrophic factor (BDNF) compared to WT mice and Montelukast (a CysLT_1_R antagonist)-treated mice also reduced BDNF expression in a mouse xenograft model with human SW480 CC cells. CD66b and BDNF expression was significantly higher in patient tumor tissues than in the matched normal mucosa. The univariate Cox PH analysis yielded CD66b and BDNF as an independent predictor of overall survival, which was also found in the public TCGA-COAD dataset. We also discovered a strong positive correlation between CD66b, BDNF and CysLT_1_R expression in the Malmö CC cohort and in the TCGA-COAD dataset. Our data suggest that CD66b/BDNF/CysLT_1_R expression as a prognostic combined biomarker signature for CC patients.

## 1. Introduction

There is growing evidence suggesting that chronic inflammation contribute significantly to the development of various types of cancers, including colorectal cancer (CRC) [1]. Colitis-associated cancer (CAC) is a type of colon cancer (CC) that is preceded by clinically detectable inflammatory bowel diseases, such as Crohn’s disease and ulcerative colitis. It has been shown that inflammatory bowel disease ranks as the third-highest risk factor for CC, behind familial adenomatous polyposis (FAP) and hereditary nonpolyposis colorectal cancer syndrome [2].

There are various similar features among CAC and sporadic CC, e.g., chromosome instability, microsatellite instability, KRAS and BRAF mutations, oncogene activation, which in turn initiates the allele removal of p53, and mutation of the tumor suppressor gene adenomatous polyposis coli (APC) [3]. In sporadic CC, following mutation of the *APC* gene, the WNT/β-catenin signaling pathway is activated, and a number of potential oncogenic target genes lead to progression from adenoma to carcinoma [4].

Despite major advances in surgical and pharmacological treatments targeting cancer, CRC still has a high mortality rate and ranks as the third leading cause of cancer-related death worldwide after lung and breast cancer. CRC is a heterogeneous disease and it is closely associated with genetic and environmental factors [5].

Polymorphonuclear neutrophils (PMNs) are often found in high numbers in the tumor microenvironment and account for 50–70% of all leukocytes in human blood [6]. PMN have a dual function in the tumor microenvironment. They fight tumor cells by releasing antimicrobial and cytotoxic content from their granules or by secreting immune mediators to activate other immune cells. However, tumor-derived factors can change PMNs into a more pro-tumor phenotype as discussed in a recent review by Mantovani and co-workers about the diversity and plasticity of neutrophils in the tumor progression [6].

Recent studies have shown that biologically significant amounts of cysteinyl leukotrienes (LTC_4_, LTD_4_ and LTE_4_) are formed after interactions between PMNs and endothelial cells [7] and contribute to changes in the microenvironment [8]. PMNs also produce LTB_4_, which has high chemo-attractive properties that can further influence the tumor microenvironment. Our previous research has shown that increased levels of CysLT_1_R (the high affinity receptor for LTD_4_) and decreased levels of CysLT_2_R (the high affinity receptor for LTC_4_) in patient tumor tissues are associated with poor prognosis for patients with CRC [9]. We have also recently reported that CysLT_1_R signaling drives 5-FU drug resistance via β-catenin in CC cells [10].

Brain-derived neurotrophic factor (BDNF) is a member of the nerve growth factor family that contributes to the differentiation, maturation, and survival of nerve cells in the nervous system [11]. BDNF has been shown to be expressed in many different solid tumors, including CC [12]. BDNF is the main ligand of tropomyosin receptor kinase B (TrkB), which is a tyrosine kinase receptor, and activation of the receptor causes phosphorylation and activation of downstream signaling pathways such as the RAS/MAPK and PI3K/AKT pathways. This contributes to tumor progression and is associated with poor prognosis [13]. A recent study reported that BDNF expression is regulated by the Wnt/β-catenin signaling pathway and that the BDNF pathway may crosstalk with the Wnt/β-catenin pathway [14]. Furthermore, in vivo studies indicated that TrkB inhibition sensitizes CRC cells to anoikis and protects against metastasis in xenograft mouse models by blocking TrkB with either Trk inhibitors or by downregulating with short hairpin (sh) RNA, leading to a reduction in both the size and number of tumors [15].

The main goal of CRC therapy is to develop better strategies to reduce the risk of tumor metastasis. Our current study is focused on the BDNF/CysLT_1_R axis as an independent prognostic marker for the OS of CC patients.

## 2. Materials and Methods

### 2.1. Patient Samples

**Figure 1:**
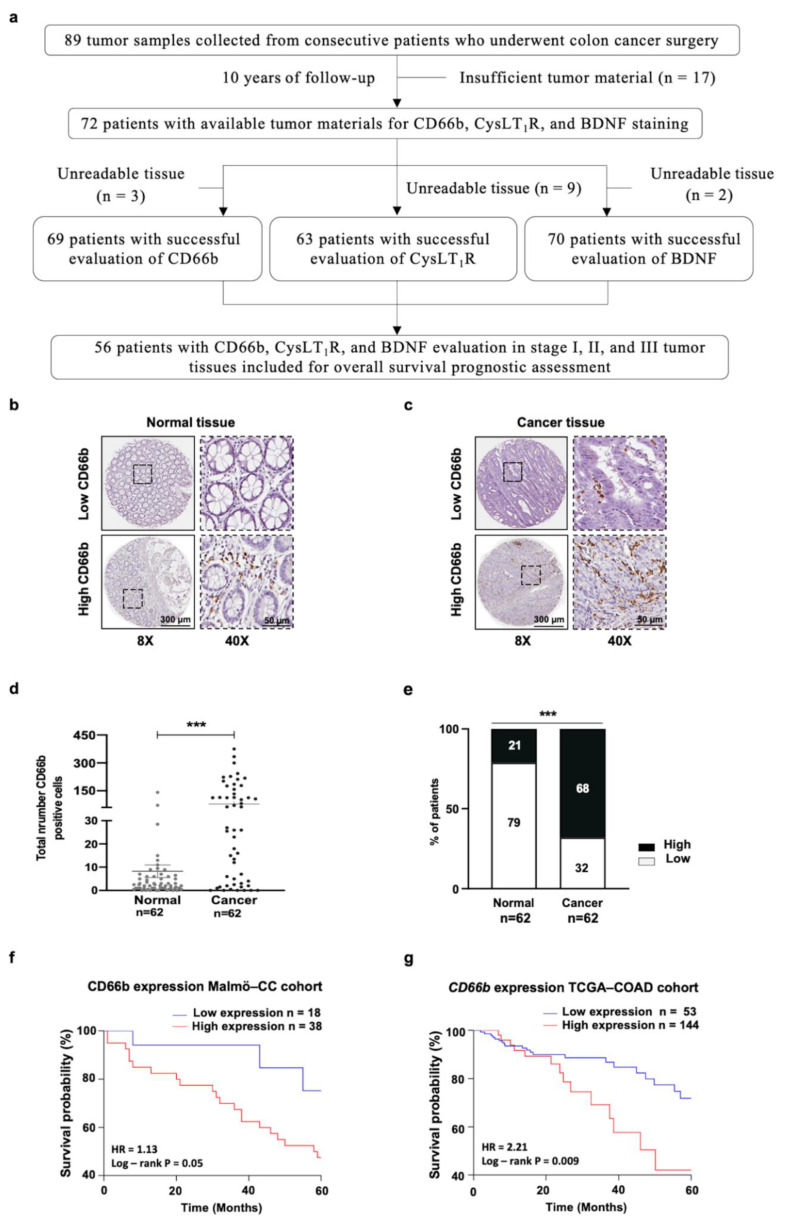
Elevation of tumor-infiltrating neutrophils in patients with colon cancer (CC). Tumor-infiltrated neutrophil valuation in tissue microarray of 72 patients with CC. (**a**) The flow chart of the study design, inclusion, and exclusion criteria. (**b**,**c**) Representative immunohistochemical staining of CD66b positive (CD66b^+^) cells showing normal (**b**) and cancer (**c**) areas with low and high tumor infiltrated CD66b^+^ neutrophils in matched normal (left) and cancerous tissue (right). Scale bars, 300 µm and 50 µm; 8× and 40× magnification. (**d**) Number of infiltrated neutrophils in normal and tumor tissue from patients with colon cancer. (**e**) Percentage of patients with low (<8) and high (≥8) tumor infiltrated neutrophil CD66b^+^ cells. Kaplan–Meier analysis with the log-rank test of overall survival (OS) stratified for CD66b^+^ cells in patients with colon cancer in Malmö CC cohort (**f**) and TCGA-COAD cohort (**g**) (cut-off based on the ROC-Youden Index). The results are shown as paired *t*-tests, (**d**) with the mean ± standard error of the mean (SEM), and (**e**) chi-square test. *** *p <* 0.001.

Tumor materials were retrospectively collected from consecutive patients who underwent CC surgery during 1990 as described before [16]. Tissue microarrays (TMAs) from formalin-fixed and paraffin-embedded CC samples and matched normal mucosa samples collected from these patients obtained from the Department of Pathology archives were used. Tissues from 72 patients with CC were used in this study see Figure 1a and their clinicopathological details, see Supplementary Table S1.

### 2.2. Immunohistochemistry (IHC)

The paraffin-embedded samples were cut into 4 µm sections, deparaffinized, and incubated in 10 mM citrate buffer (pH 6.0, S1699; Dako, Glostrup, Denmark) for 20 min in a microwave oven. Blocking buffer (Dako) with 10% fetal bovine serum in PBS was added to the slides for 10–20 min, and then a specific serum-free protein block (Dako) was added for 30 min at RT. After washing, the sections were stained using Dako Autostainer Plus [16] with the following primary antibodies: rat monoclonal anti-CD66b antibody (1:500; ab233811, clone 2F12E8; Abcam, Cambridge, United Kingdom), rabbit monoclonal anti-BDNF (CRC patients 1:500, for mice 1:1000, pH 6, RT, 1 h; ab108319, Abcam). The secondary antibody Envision+ System-HRP-labelled polymeric anti-rat/anti-rabbit antibody (Dako) was visualized using 3,3′-diaminobenzidine (DAB) substrate (Vector Laboratories Inc., Burlingame, CA, USA) and counterstained with hematoxylin.

### 2.3. Quantitative Real-Time PCR

Total RNA was isolated from collected tumor tissue (*n* = 10) and its matched normal area (*n* = 10) of colon cancer patients, using Qiagen RNeasy Plus Mini Kits (Qiagen, Hilden, Germany) according to the manufacturer’s protocol. The Revert Aid H Minus First Strand cDNA Synthesis Kit (Invitrogen, Carlsbad, CA, USA) was used to prepare cDNA synthesis. Real-time PCR was performed using the following TaqMan primers: *CYSLTR1* (Hs 00272624_s1) and *BDNF* (Hs 02718934_s1). Amplifications were performed in a Stratagene Mx 3005P system (Agilent Technologies, Inc., Santa Clara, CA, USA). Reactions were normalized to the *HPRT1* (Hs99999909_m1) housekeeping gene. Gene expression was assessed using the comparative threshold cycle (Ct) method and analyzed with MxPro qPCR software (Agilent Technologies, Santa Clara, CA, USA).

### 2.4. Colitis Associated Colon Cancer Mouse Model

The *cysltr1* gene disrupted (*cysltr1^−/−^*) C57BL/6N mice were a gift from Prof. Frank Austen (Harvard Medical School, Brigham and Women’s Hospital, Boston, MA, USA) as described previously [17,18]. These mice were bred and maintained at the animal facility in accordance with ethical permit, approved by the Regional Ethics Committee for Animal Research. The AOM-DSS mouse model was established as described previously [19]. The size and number of colonic tumors were evaluated using a dissection microscope (20×), and the colon was finally subdivided into smaller pieces (approx. 2 cm) and embedded in paraffin for IHC analysis. The IHC evaluation of colon sections were performed by evaluating six random areas of the whole colon. The calculated mean values were compared between the genotypes.

### 2.5. Mouse Xenograft Study

Female nude mice (BalbC nu/nu; 5–6 weeks old) were used, and animal experiments were approved by the Regional Ethics Committee for Animal Research. The xenografts were induced by subcutaneous injection of 2.5 × 10^6^ low-passage human SW480 colon cancer cells into both flanks of the mice [20]. Tumor development was monitored by palpation. Once palpable tumors were detected, the mice were randomly divided into two groups, treated with either vehicle (DMSO) or Montelukast (Mo), a CysLT_1_ antagonist. The mice received daily i.p. injections of Mo (5 mg/kg) or DMSO. After 21 days, the mice were sacrificed, and the tumors were removed. The tumor xenograft tissues were processed for IHC or Western blot analysis. The whole xenograft sections were evaluated and the calculated mean was compared between the vehicle treated control group and the Mo treated group. For Western blot analysis, total protein was extracted from DMSO (*n* = 4) and Mo (*n* = 4) treated mice tumor tissues. Protein was transferred to PVDF membranes (Bio-Rad, Hercules, CA, USA), and blocked with 5% BSA in TBST with 0.5% Tween-20 (A4974, PanReac AppliChem, Darmstadt, Germany) and incubated with specific primary rabbit monoclonal anti-BDNF antibody (1:1000; ab108319; Abcam, Cambridge, UK) or CysLT_1_R antibody (1:1000; NBP2-92396; Novus Biologicals Littleton, Colorado, USA) overnight at 4°C. Thereafter the membranes were washed four times and incubated with secondary antibody (goat anti-rabbit IgG; 1:1000; Ab205718; Abcam) for 1 h at room temperature. GAPDH (1:1000; sc-25778; Santa Cruz Biotechnology, Santa Cruz, CA, USA) was used as the loading control. Protein expression was visualized by Immobilon Western Chemiluminescent HRP Substrate (Immobilon Western, Merck Millipore, Billerica, MA, USA) and detected using the Bio-Rad ChemiDoc™ Imaging system. Western blot quantification was performed by Image J software. 

### 2.6. Acquisition of Gene Expression and Clinical Data from The Cancer Genome Atlas (TCGA) Dataset

Normalized RNA-sequencing data as transcripts per million (TPM) and the associated clinical information of the COAD samples were downloaded from The Cancer Genome Atlas (TCGA) dataset (https://portal.gdc.cancer.gov/; https://tcpaportal.org/tcpa/; 20 June 2020). Out of 315 CC cases, 22 cases missing pathological information, 21 cases with a follow-up period of ≤30 day, 46 cases with metastasis (Stage IV), and 29 cases which did not have any of the genes of interest were eliminated. Thus, 197 (stage I, II and III) cases and clinical information were included in the study. Normalized gene expression data for the TCGA-COAD dataset were log2-transformed for further analysis.

### 2.7. Identification of Independent Prognostic Parameters of Colon Cancer

To identify and validate the independent prognostic value of *CD66b*, *BDNF*, and *CYSLTR1* at mRNA level, univariate and multivariate Cox regression analyses were performed in the TCGA-COAD dataset. Parameters with *p* < 0.05 based on the univariate analysis were further included in the multivariate Cox regression analysis (adjusted for age, LNM and TNM stage). TCGA samples were divided into high- and low-risk groups according to the optimal cut-offs determined by the ROC-Youden Index association criteria.

### 2.8. Statistical Analysis

The results are expressed as the mean ± standard error of the mean (SEM). All comparisons between the mean values were performed using Student’s *t*-test and categorical variables were compared with chi-square test. Survival curves, which were generated via the Kaplan–Meier method, were compared using the log-rank test. To evaluate the association of protein expression in CC tissue with OS, univariate and multivariate Cox proportional hazards regression models were applied, and hazard ratios (HRs) together with 95% confidence intervals (CIs) were calculated to determine the risk of death. The multivariate model was adjusted for established prognostic factors such as age, gender, lymph node metastasis (LNM), tumor-node-metastasis (TNM) stage and tumor size. All patients with incomplete or missing cores were excluded from the analysis. Finally, we used receiver operating characteristic (ROC) curves and calculated the area under the curve (AUC) to determine the predictive ability of the final model compared to the unadjusted and adjusted model. Statistical analyses were performed using MedCalc version 19.3 (MedCalc Software Ltd., Ostend, Belgium), SPSS version 23.0 (SPSS, IBM, Armonk, NY, USA) and GraphPad Prism version 8.0a (GraphPad Software, Inc., San Diego, CA, USA). A two-sided *p* value < 0.05 was considered statistically significant. For the association study of high or low status of the *CD66b*, *BDNF*, and *CYSLTR1* genes, clinicopathological parameters were analyzed and presented as a Circos plot using Circos software.

## 3. Results

### 3.1. Elevation of Tumor-Infiltrating Neutrophils in Patients with Colon Cancer 

The tumor microenvironment is complex, and the role of tumor-infiltrating neutrophils is not yet fully understood. To evaluate the role of tumor-associated neutrophils, we started by staining the Malmö CC patient cohort against the neutrophil marker CD66b. Sixty-two patient samples of colon cancer tissues and matched normal mucosa were evaluated by immunohistochemistry (IHC) (Figure 1a). The majority of patients with CC had been diagnosed with low differentiation grade tumors (72%), and only a few had distant metastasis (19%). The clinicopathological characteristics of the patients are described in Supplementary Table S1. Representative CD66b IHC staining of normal mucosa and its matched tumor area are shown (Figure 1b,c). A significantly increased number of CD66b^+^ cells was found in the tumor tissue area compared to the normal tissue area (Figure 1b–e). Neutrophil infiltration varied widely in the tumor tissues, and we found a significant difference between normal mucosa with a mean of 8/core and tumor tissue with a mean of 225/core, *p* < 0.001 (Figure 1d). We next investigated the distribution of neutrophils in the tumor tissues and in the normal matched pair area. The mean number of infiltrated neutrophils in the normal colon tissues (*n* = 8) and the cut off was calculated based on the ROC curve analysis of infiltrated neutrophil CD66b^+^ cells in tumor and normal tissues which determined the high and low CD66b^+^ (neutrophil) groups (Figure 1e). We found a statistically significant difference between normal and cancerous areas, with 68% high number of CD66b^+^ neutrophils in the tumor area compared to only 21% in the normal area (*p* < 0.001; Figure 1e). We next performed a Kaplan–Meier overall survival (OS) analysis of infiltrated neutrophils in tumor tissue between the low and high CD66b^+^ groups. A significant correlation was found between the two groups in the Malmö CC patient cohort with stage I-III (Figure 1f). High CD66b^+^ group showed poorer prognosis compared to the low CD66b^+^ group (HR = 1.13, *p* = 0.05) in Malmö CC patient cohort. Interestingly, we found a significant difference in patients with stage I-III CC as well as in the TCGA cohort of patients with colon cancer (HR = 2.21, *p* = 0.009; Figure 1g). These data indicate that high neutrophil infiltration correlates with shorter OS compared to those with low neutrophil infiltration (Figure 1f,g). These data indicate that neutrophils can have an effect on tumor progression and encouraged us to further investigate the impact of neutrophils.

### 3.2. BDNF and CysLT_1_R Positively Correlates in Colon Cancer Patients

**Figure 2:**
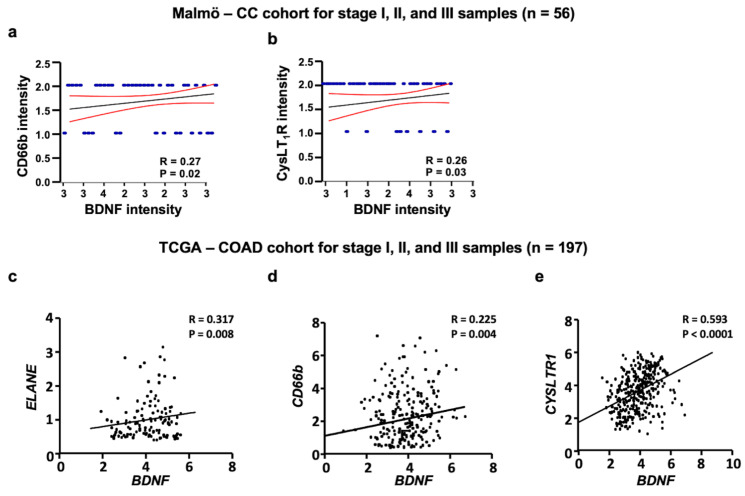
BDNF and CysLT_1_R positively correlates in colon cancer patients. (**a**,**b**) Spearman Correlation of BDNF expression with (**a**) CD66-positive (CD66b^+^) neutrophil expression and (**b**) CysLT_1_R. (**c**–**e**) XY scatter plot of *BDNF* mRNA levels with (**c**) *ELANE*, (**d**) *CD66b* and (**e**) *CYSLTR1* in TCGA-COAD cohort.

We next explored the relation between BDNF, neutrophils, and CysLT_1_R in colon cancer tissue. Interestingly, we found a positive correlation between BDNF protein expression and CD66b (neutrophil) protein expression in Malmö CC cohort (*p* = 0.02; Figure 2a) as well as with CysLT_1_R protein expression (*p* = 0.03; Figure 2b). We next investigated the association between *BDNF* and neutrophils, identified as *CD66b* or *ELANE* (neutrophil elastase). The TCGA dataset with 197 patients with CC in stages I, II and III was used and showed a significant positive correlation of *BDNF* expression with *ELANE* (*p* = 0.008), *CD66b* (*p* = 0.004), and *CYSLTR1* (*p* < 0.0001) (Figure 2c–e, respectively).

### 3.3. Functional Absence of CysLT_1_R Negatively Regulates BDNF Expression

**Figure 3:**
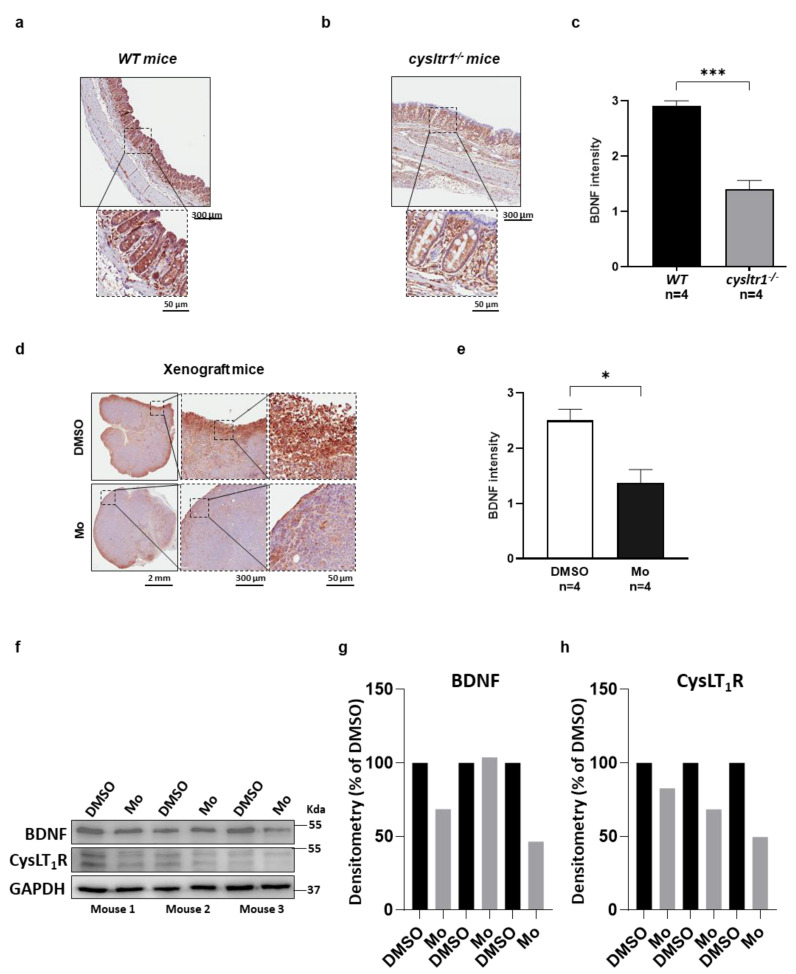
Functional absence of CysLT_1_R negatively regulates BDNF expression. BDNF protein expression in mouse colon tissues of colitis-associated carcinoma (CAC; AOM/DSS-treated) mice. Immunohistochemistry evaluation in colon sections were performed by evaluating six random areas in the whole colon. Representative images (8× and 40× magnification) showing one out of six areas from (**a**) wild-type (*WT*) mice (*n* = 4) and (**b**) *cysltr1* gene disruption (*cysltr1^−/−^*) mice (*n* = 4) and (**c**) the corresponding bar diagram showing BDNF intensity. (**d**) The mice xenograft sections were evaluated for BDNF intensity. Representative images showing the mouse xenograft section for the group treated either with vehicle control (DMSO) or the CysLT_1_R antagonist, Montelukast (Mo), and (**e**) the corresponding bar diagram. Western blot and densitometry analyses of BDNF (**f**,**g**) and CysLT_1_R (**f**,**h**) protein expression in mice xenograft tumor tissues from DMSO vehicle control or Mo treated mice. Scale bars as indicated in the images. * *p <* 0.05, *** *p <* 0.001.

To evaluate whether BDNF expression is affected by the absence of CysLT_1_R, we used a mouse model with a disrupted *cysltr1* gene (*cysltr1^−/−^*) [18,19]. We first stained with the anti-Ly6G antibody (a mice neutrophil marker) colon tissues from wild-type mice (WT, *n* = 4) and *cysltr1^−/−^* mice (*n* = 4), to evaluate the difference between WT and *cysltr1^−/−^* mice. However, no significant difference between the WT and the *cysltr1^−/−^* mice was found for Ly6G^+^ cells (neutrophils), see Supplementary Figure S1a,b. We next stained these mice colon tissues from WT and *cysltr1^−/−^* mice with the BDNF antibody. A representative image of WT mouse colon showing high BDNF expression (Figure 3a), and a representative image of *cysltr1^−/−^* mouse colon showing lower BDNF expression (Figure 3a). We found a significant downregulation of BDNF expression in *cysltr1^−/−^* mouse colon (*p* < 0.001; Figure 3c, see Supplementary Figure S2a for the other three WT and *cysltr1^−/−^* mouse colons BDNF expression). We next used a mouse xenograft model with SW480 cells with or without treatment with the CysLT_1_R specific antagonist Montelukast (Mo) [20] (Figure 3d and Supplementary Figure S2b), which is used in the clinic for patients with asthma. We found a significant downregulation of BDNF expression in Mo-treated mice compared to control mice (*n* = 4; Figure 3e). Furthermore, we also found a significant downregulation of BDNF and CysLT_1_R expression in Mo treated group compared to the DMSO treated control mice by Western blot analysis (*n* = 3; Figure 3f–h). 

### 3.4. Expression Levels of BDNF in Patient Colon Cancer Tissues Negatively Correlated with CC Patient Survival

**Figure 4:**
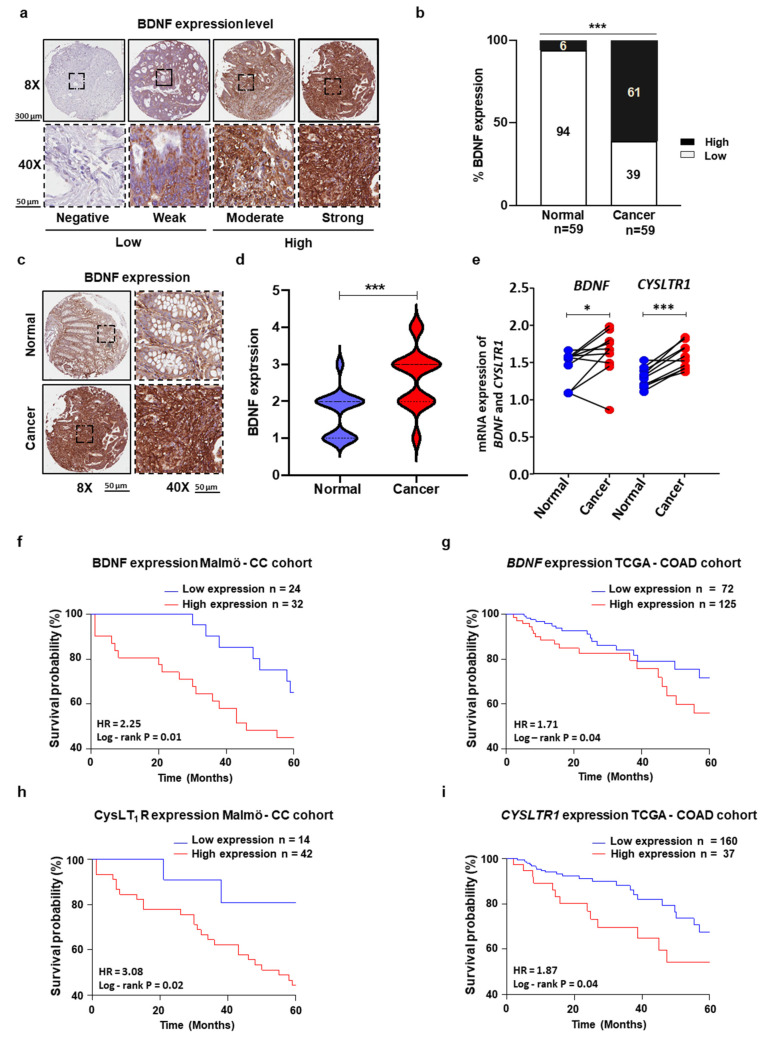
Expression levels of BDNF in patient colon cancer (CC) tissues negatively correlated with CC patient survival. (**a**) Representative BDNF IHC staining of the CRC patient cohort with negative, weak, moderate, and strong BDNF expression levels, n = 59; 8× (scale bars, 300 μm) and 40× (scale bars, 50 μm) magnification. (**b**) Percentage of patient tissues with low (negative, weak) and high (moderate, strong) BDNF expression. (**c**) Representative BDNF IHC staining of normal colon tissue (upper images) and matched colon tumor tissues (lower images). (**d**) IHC scoring of the level of BDNF intensity shown by the violin plot from normal areas and the matched tumor area. (**e**) qPCR analysis showing the expression of *BDNF* and *CYSLTR1* in matched paired normal mucosa and cancer tissue samples from 10 CRC patients. (**f**,**g**) Kaplan–Meier survival curves in Malmö CC cohort (**f**) and TCGA-COAD cohort (**g**) according to the expression status of BDNF using the log-rank test. (**h**,**i**) Kaplan–Meier survival curves in Malmö CC cohort (**h**) and TCGA-COAD cohort (**i**) according to the expression status of CysLT_1_R using the log-rank test. The results are shown as the mean ± standard error of the mean (SEM), paired *t*-test (**d**) and chi-square test (**b**). Scale bars as indicated in the images. * *p <* 0.05, *** *p <* 0.001.

To investigate the importance of BDNF expression in CC, we stained our CC cohort with patient tumor samples and their matched corresponding normal mucosa with the BDNF antibody. We found 56 patient tumor and its matched normal mucosa and representative BDNF staining is shown for normal mucosa and tumor areas in Figure 4a–d. BDNF expression was significantly increased in tumor areas compared to matched normal tissues, with an IHC mean score of 2 for normal tissue and a mean score of 3 for tumor tissue (Figure 4d). We next categorized BDNF tumor expression into two groups: low (negative and weak, found in 24 patients) and high (moderate and strong, in 32 patients). We found high BDNF expression in 61% of the patient tumor tissues, while high BDNF expression in matched normal tissues was observed in only 6% (Figure 4b). Next, we examined the mRNA expression of *BDNF* and *CYSLTR1* in tumor and its matched normal tissue samples from CRC patients and the qRT-PCR analysis significantly showed upregulation in tumor samples compared to its normal tissue samples for both genes (Figure 4e).

Next, to determine the influence of BDNF on stage I, II and III CC patient overall survival, we used the two groups of patients with low and high levels of BDNF. The Kaplan–Meier survival analysis and log-rank test revealed that patients with high BDNF expression exhibited poor overall survival (OS) compared to patients with low BDNF expression (HR = 2.25, *p* = 0.01; Figure 4f). Furthermore, we found similar result in TCGA-COAD cohort with stage I, II and III patients (HR = 1.71, *p* = 0.04; Figure 4g). We have observed the univariate analyses for each potential predictor with respect to OS using the Cox regression approach showed a significant association with BDNF (HR = 1.42, 95% CI = 0.79–2.54, *p* = 0.02, Supplementary Table S2).

### 3.5. A Potential Role for BDNF/CysLT_1_R as a Prognostic Predictor of Colon Cancer Development

**Figure 5:**
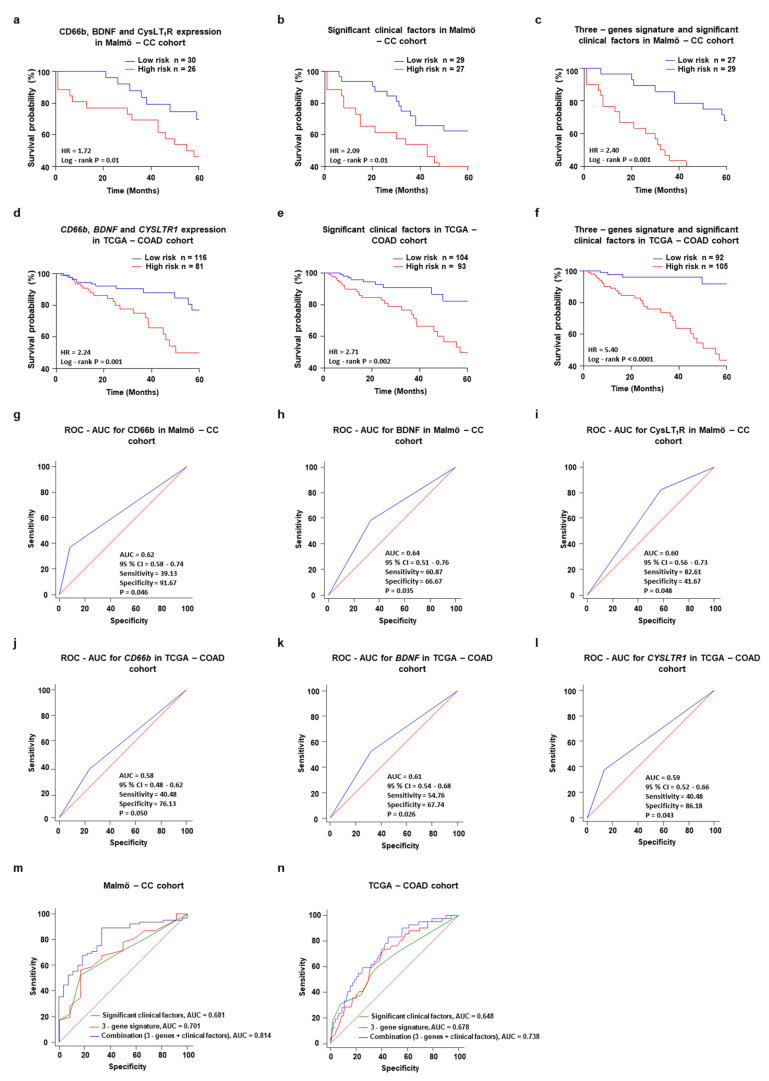
A potential role for BDNF/CysLT_1_R/CD66b as a prognostic predictor of colon cancer development. Prognostic and predictive analysis of CD66b, BDNF and CysLT_1_R protein and gene signatures in patients with colon cancer. (**a**) Kaplan–Meier survival curves according to the three-protein signature (CD66b, BDNF, and CysLT_1_R) expression in subsets of patients with colon cancer. (**b**) Kaplan–Meier survival curves according to significant clinical factors (gender, lymph node metastasis, TNM staging) from univariate analysis in Malmö CC patient cohort (**c**) Multivariate analysis of the three-protein signature with the significant clinical features (gender, lymph node metastasis and TNM staging) in Malmö CC patient cohort, cut-off based on the Youden Index = 0.38, association criteria >3.11. (**d**–**f**) Kaplan–Meier survival curves according to (**d**) three-gene signature (*CD66b*, *BDNF* and *CYSLTR1*) expression (**e**) significant clinical factors (age, lymph node metastasis and TNM staging) from univariate analysis and (**f**) multivariate analysis of the three-genes signature with the significant clinical features (gender, lymph node metastasis and TNM staging) in TCGA-COAD patient cohort, cut-off based on the Youden Index = 0.39, association criteria >1.91. (**g**–**l**) ROC curves to determine the predictive ability of (**g**) CD66b, (**h**) BDNF, (**i**) CysLT_1_R expression in Malmö CC patient cohort, and (**j**) *CD66b*, (**k**) *BDNF*, (**l**) *CYSLTR1* in the TCGA–COAD cohort. (**m**,**n**) A summary of the ROC curve and prognostic value determination regarding colon cancer clinical features in (**m**) Malmö CC and (**n**) TCGA-COAD patient cohort.

Previously, we have reported that high CysLT_1_R expression is associated with poor prognosis for patients with colon cancer [9]. In addition, Moore and co-workers have suggested a crosstalk between cancer cells and the 5-lipoxygenase pathway [21]. As previously shown, high CysLT_1_R expression in tumor tissues was associated with poor prognosis for CC patients in Malmö CC cohort (HR = 3.08, *p* = 0.02; Figure 4h) and TCGA-COAD cohort (HR = 1.87, *p* = 0.04; Figure 4i). We further evaluated the best fit multivariate Cox models using the three-protein signature (CD66b, CysLT_1_R, BDNF) in tumor tissues from the univariate model to better predict OS and separate the high-risk patient group (HR = 1.72, *p* = 0.01; Figure 5a). Therefore, we next preformed Cox regression analysis of the Malmö CC patient cohort which showed that gender, TNM and LNM were significantly associated with overall survival of the stage I, II and III patients (Supplementary Table S2). The Kaplan–Meier survival analysis with these three factors significantly separate the high-risk patients group (HR = 2.09, *p* = 0.01; Figure 5b). As previously mentioned, these factors are usually applied to estimate the prognosis of CC patients [22]. We thereafter calculated the risk score of the combined expression of the three proteins and the significant clinical factors (gender, LNM and TNM) by Kaplan–Meier survival analysis in Malmö CC patient cohort, stage I-III (HR = 2.40, *p* = 0.001; Figure 5c). The *CD66b*, *BDNF* and *CYSLTR1* association was also found in the TCGA-COAD dataset (Figure 5d–f). As additional information, different stages of the tumor, according to patient’s survival were discriminated and the results shown in Supplementary Figures S3 and S4.

We next created receiver operating curves (ROCs) for CysLT_1_R and BDNF alone as well as combined, all with significant results. The CD66b, CysLT_1_R and BDNF ROC curves presented a clear positive value and high accuracy in predicting patients at high risk or with a poor prognosis based on the individual high expression of CD66b (AUC = 0.62, *p* = 0.046, specificity = 91.67% and sensitivity = 39.13%; Figure 5g), BDNF (AUC = 0.64, *p* = 0.035, specificity = 66.67% and sensitivity = 60.87%; Figure 5h) and CysLT_1_R (AUC = 0.60, *p* = 0.048, specificity = 41.67% and sensitivity = 82.67%; Figure 5i). We have observed similar result for the ROC analysis of *CD66b*, *BDNF* and *CYSLTR1* mRNA expression in TCGA-COAD dataset (Figure 5j–l). The proposed marker sensitivity and specificity for combined CD66b, CysLT_1_R and BDNF is the best model with more specificity for detecting high-risk patients (AUC = 0.70). We next estimate the ROC-AUC value with the best fit multivariate Cox models using CD66b, BDNF, CysLT_1_R expression in tumor tissues and the significant clinical factors (gender, LNM, TNM) from the univariate model. The combination model was a better predictor for OS with a higher AUC value (AUC = 0.81; specificity = 83.33% and sensitivity = 80.52%; Figure 5m) than the independent predictors of CD66b (AUC = 0.62), BDNF (AUC = 0.64), CysLT_1_R (AUC = 0.60) and the combination of CD66b, BDNF and CysLT_1_R expression (AUC = 0.70, Figure 5m) was seen in colon cancer patients. The ROC analysis result was also convincing in TCGA-COAD dataset and the best fit multivariate Cox models using *CD66b*, *BDNF*, *CYSLTR1* expression in tumor tissues and the significant clinical factors (age, LNM and TNM) was achieving high AUC values (AUC = 0.73, Figure 5n). The combination of CD66b, BDNF, and CysLT_1_R was shown to be useful for detecting high-risk colon cancer patients.

### 3.6. CD66b Neutrophil, BDNF and CysLT_1_R Expression with Significant Clinicopathological Factors Impact Patient Survival Probability 

**Figure 6:**
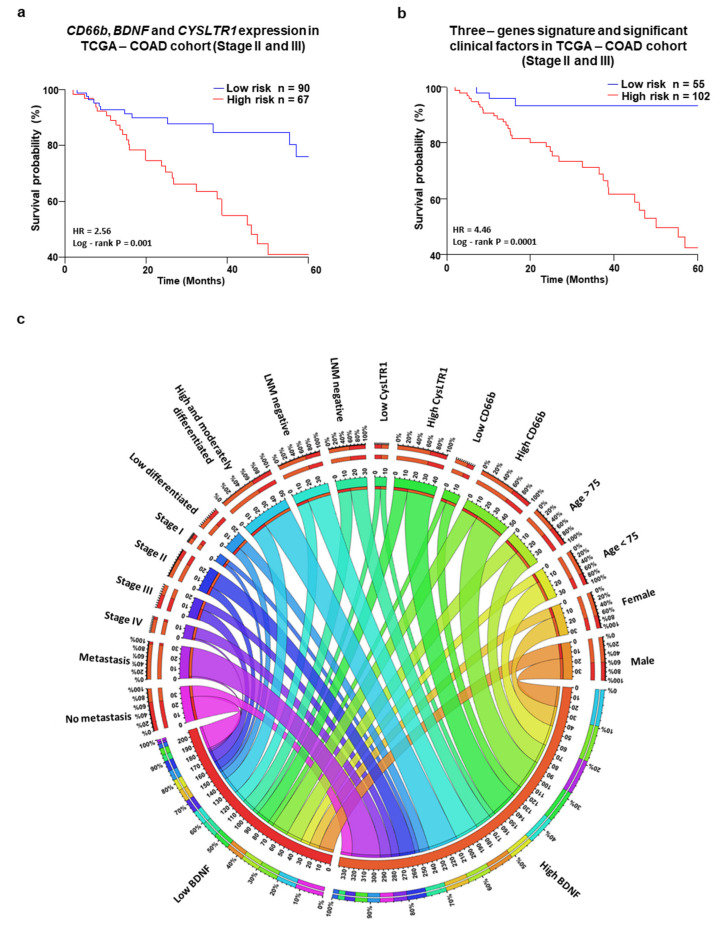
CD66b neutrophil, BDNF and CysLT_1_R expression with significant clinicopathological factors impact patient survival probability. Kaplan–Meier survival curves according to (**a**) three-gene signature (*CD66b*, *BDNF* and *CYSLTR1*) expression and (**b**) multivariate analysis of the three-gene signature with the significant clinical features in stage II and III in TCGA-COAD patient cohort. (**c**) Circos plot showing the association between low and high BDNF expression and tumor stage, lymph node metastasis, tumor size, sex and age group involved in colon cancer, and the number of metastatic patients at diagnosis. The frequency of occurrence of different factors, such as BDNF protein expression pattern and tumor stage, is depicted in the outer ring. The inner ring of the Circos plot depicts the association between the BDNF protein expression pattern and tumour stages. The thickness of each color shows the frequency of correlation with factors specifically related to BDNF protein expression.

Using the TCGA-COAD dataset, we further analyzed the impact of *CD66b*, *BDNF* and *CYSLTR1* without or with the significant clinical factors in stage II and III patients (Figure 6a,b), and both the conditions found significant. Finally, a summary of the association of high and low expression of BDNF, CysLT_1_R, and CD66b (neutrophils) expression and clinicopathological factors is shown in the Circos plot (Figure 6c). Our results suggests that high CD66b neutrophil content in the tumor microenvironment increase the BDNF production via CysLT_1_R signaling, which led to poor survival of patients with CRC.

## 4. Discussion

Our results indicate that high tumor-infiltrating neutrophils in patients with colon cancer is associated with poor prognosis and that the tumor associated neutrophils (TAN) can be the source of BDNF production in the TME, where BDNF can further accelerate tumor progression. We identified the CD66b/BDNF/CysLT_1_R axis as an independent prognostic marker for OS in CC patients and integrated with clinical factors as a strong predictor. There are contrary reports concerning tumor-infiltrating neutrophils and the prognosis of human CC patients. Some studies have reported that CC patients with high neutrophil infiltration show a significantly better prognosis related to their survival [23], while other studies have derived opposite conclusions: increased intra-tumor neutrophils in CC may cause a worse prognostic phenotype [24]. Based on this knowledge, adjuvant chemotherapy can induce neutropenia and affect the outcomes of patients with CC. Our CC patient cohort is suitable for this type of study because they did not receive any adjuvant chemotherapy before tumor resection due to the time of surgery at that time. We evaluated the appearance of CD66b-positive neutrophils by IHC analysis and found that CD66b-positive neutrophils were significantly upregulated in tumor tissues compared to matched normal tissues. Interestingly, we found a significant correlation in patients with stage I, II and III CC in which patients with high CD66b expression had poorer survival than patients with low CD66b expression levels in Malmö CC cohort and at gene level in the TCGA-COAD dataset. These results suggest that high levels of neutrophils are associated with a poorer prognosis for CC patients. According to previous reports, interaction and signal exchange between neutrophils and the TME play a crucial role in tumor progression or suppression [25,26].

BDNF plays an important role in neuronal survival but is also involved in various cancers and is expressed by a variety of peripheral tissues and immune cells, such as eosinophils and neutrophils [27]. As early as the 1990s, the effect of nerve growth factor (NGF) on survival, phagocytosis, and superoxide production was examined in murine neutrophils isolated from peripheral blood and the abdominal cavity, and it was reported that NGF prolonged the survival of neutrophils and enhanced phagocytosis of hydrophilic microspheres by peritoneal neutrophils [28]. Another study recently reported a higher level of BDNF protein in neutrophil rich sputum from patients with asthma than in sputum from patients with lower neutrophil counts [29]. However, the biological function and mechanism of action of BDNF in cancer progression are still unclear.

To distinguish between low and high BDNF expression related to survival with sufficient contrast and statistical power, we then determined whether *BDNF* was expressed in a larger dataset (TCGA-COAD) derived from 197 patients with colorectal cancer. mRNA data showed longer survival in patients with low *BDNF* expression than in those with high *BDNF* expression. These public colon cancer datasets showed a statistically significant association between *BDNF* and two neutrophilic markers, *CD66b* and neutrophil elastase (*ELANE*).

Further evidence indicates that tumors surrounded by inflammatory cells in the TME might have increased expression of BDNF. Furthermore, our IHC analysis of the CC cohort showed that high BDNF expression was significantly upregulated in colon cancer tissues, which was correlated with poor survival. Previously, shown that low CysLT_1_R expression in patients with colon cancer is associated with better survival and that high levels are associated with poor prognosis [9,10,30]. In this study, we investigated the predictive value of CD66b, BDNF and CysLT_1_R expression regarding patient overall survival. Interestingly, we found that patients with both low CD66b, BDNF and CysLT_1_R expression had significantly better survival than patients with high expression levels of CD66b, BDNF and CysLT_1_R. We also investigated the clinicopathological significance of CD66b, BDNF and CysLT_1_R expression in CC patients. Previous studies have shown that the survival rate of patients with colon cancer is associated with the stage and early detection of the disease [31] and that patients with the same TNM stage can have different prognoses [32], indicating the molecular and cellular diversity of the disease, which may emphasize the need for better tumor markers to provide a better target for diagnosis and treatment. Tanaka and coworkers have suggested BDNF/TrkB signaling as a potential therapeutic target for CC patients [33]. Moreover, we also found a positive correlation between CD66b, CysLT_1_R, BDNF with TNM and LNM stage in CC patients. Univariate and multivariate analyses of our data showed that CD66b, CysLT_1_R and BDNF were good prognostic factors independent of clinical factors, such as gender, TNM and LNM stage. Our results provide evidence that the combination of CD66b, CysLT_1_R and BDNF expression in CC identifies a CC patient group with poor prognosis.

Mouse studies have shown augmented expression of BDNF at both the protein and mRNA levels in response to allergic reactions [31]. Here, we show that the expression of BDNF is reduced in mice lacking the *cysltr1* gene, showing the involvement of CysLT_1_R signaling. We also found that SW480 CC cell derived xenograft with montelukast, the CysLT_1_R antagonist, treatment exhibited a significant reduction in BDNF expression compared to the vehicle (DMSO) treated control.

## 5. Conclusions

In summary, to the best of our knowledge, the current study presents for the first time a positive correlation between BDNF and CysLT_1_R in colon cancer patients. We have also identified CD66b, CysLT_1_R and BDNF as a combined signature as an independent prognostic biomarker for CC patient survival. Incorporation of the three-genes signature axis into clinical predictors may, as a prognostic marker, contribute to identifying patients with poor prognosis.

## Data Availability

The datasets used and/or analyzed during the current study are available from the corresponding author on request.

## Supplementary Materials

The following are available online at https://www.mdpi.com/article/10.3390/cancers13215520/s1. Figure S1: Neutrophil (Ly6G+) population in mouse colon tissues of colitis-associated carcinoma (CAC; AOM/DSS-treated) mouse model, Figure S2: Functional absence of CysLT_1_R negatively regulates BDNF expression, Figure S3: Kaplan-Meier survival curves showing gene signature of (a–c) *CD66b*, (d–f) *BDNF* and (g–i) *CYSLTR1* in stage I, II, and III patient groups respectively in TCGA-COAD patient cohort, Figure S4, Kaplan-Meier survival curves according to (a–c) three-gene signature (*CD66b*, *BDNF* and *CYSLTR1*) expression from univariate analysis and (d–f) multivariate analysis of the three-gene signature with the significant clinical features (gender, lymph node metastasis and TNM staging) in stage I, II, and I patient groups in TCGA-COAD patient cohort, Table S1: Distribution of clinical and pathological covariates of 72 colorectal cancer patients, Table S2: Univariate and multivariate analysis of association of CD66b, BDNF, CysLT_1_R protein expression and clinical factors of patients 5 years overall survival.
