# Supplementary material for: Brain-Derived Neurotrophic Factor, Neutrophils and Cysteinyl Leukotriene Receptor 1 as Potential Prognostic Biomarkers for Patients with Colon Cancer
Source: Cancers (Basel). 2021 Nov 3;13(21):5520. doi: 10.3390/cancers13215520 (PMC8583027; doi:10.3390/cancers13215520)
Supplement: Supplementary file 1 [file cancers-13-05520-s001.zip › cancers-1391199-supplementary-4_Nov.pdf]

Supplementary Table 1: Distribution of clinical and pathological covariates of 72 colorectal cancer patients.

Clinicopathological parameters	Patients (n = 72)		Percentage	
**Sex**				
Male	33		46	
Female	39		54	
**Age**				
≤74	33		46	
> 74	39		54	
**Pathological stage**				
Stage I	9		13	
Stage II	28		39	
Stage III	21		29	
Stage IV	14		19	
**Lymph node involvement**				
No	40		56	
yes	32		44	
**Tumor differentiation**				
None/ low	52		81	
Medium/high	20		19	
**Metastasis at the time of diagnosis**				
No	58		81	
yes	14		19	
	**Normal**	**Cancer**	**Normal**	**Cancer**
**Neutrophil**				
Low	50	18	79	26
High	13	51	21	74
Missing	9	3	-	-
**BDNF**				
Low	45	30	92	43
High	4	40	8	57
Missing	22	2	-	-
**CysLT$_1$R**				
Low	15	12	26	21
High	42	46	74	79
Missing	15	9	-	-

Supplementary Table 2: Univariate and multivariate analysis of association of CD66b, BDNF, $CysLT_1R$ protein expression and clinical factors of patients 5 years overall survival.

Factors	HR	95% CI	P-value
**Univariate analysis**			
CD66b	0.67	0.37 – 1.23	0.02
BDNF	1.42	0.79 – 2.54	0.02
$CysLT_1R$	1.55	0.72 – 3.31	0.03
Age	1.03	0.99 – 1.08	0.08
Gender	2.12	1.18 – 3.80	0.01
Differentiation	0.86	0.40 – 1.82	0.70
LNM	1.85	1.00 – 3.39	0.04
Tumor size	2.71	1.37 – 5.35	0.07
TNM-stage	1.53	0.96 – 2.43	0.05
**Multivariate analysis**			
CD66b	0.84	0.62 – 1.57	0.03
BDNF	1.03	0.81 – 2.49	0.03
$CysLT_1R$	1.79	1.16 – 2.74	0.04
Gender	1.58	1.13 – 2.82	0.03
LNM	1.62.	1.21 – 3.59	0.08
TNM-stage	1.68	1.46 – 2.84	0.13

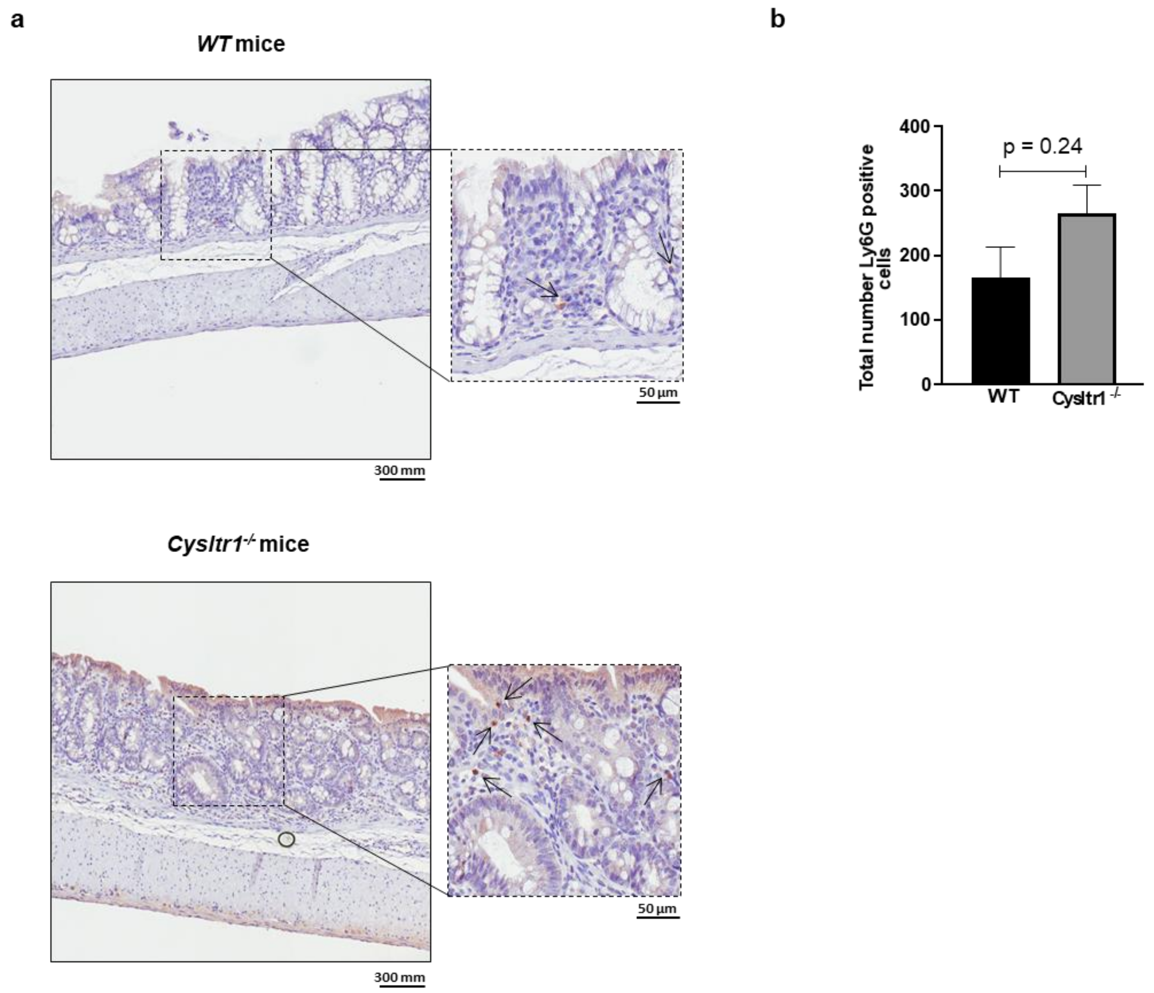

Supplementary Figure 1. Neutrophil (Ly6G$^+$) population in mouse colon tissues of colitis-associated carcinoma (CAC; AOM/DSS-treated) mouse model. Immunohistochemistry evaluation of colon sections was performed by evaluating six random areas of the whole colon. Representative images (8x and 40x magnification) showing one out of six areas from (**a**) wild-type (*WT*) mice (n = 4) and *Cysltr1* gene disruption (*Cysltr1*$^{-/-}$) mice (n = 4) and black arrows in the micrographs indicate Ly6G$^+$ cells. (**b**) The corresponding bar diagram showing total number of Ly6G$^+$ cells in each group of mice. Scale bars as indicated in the images.

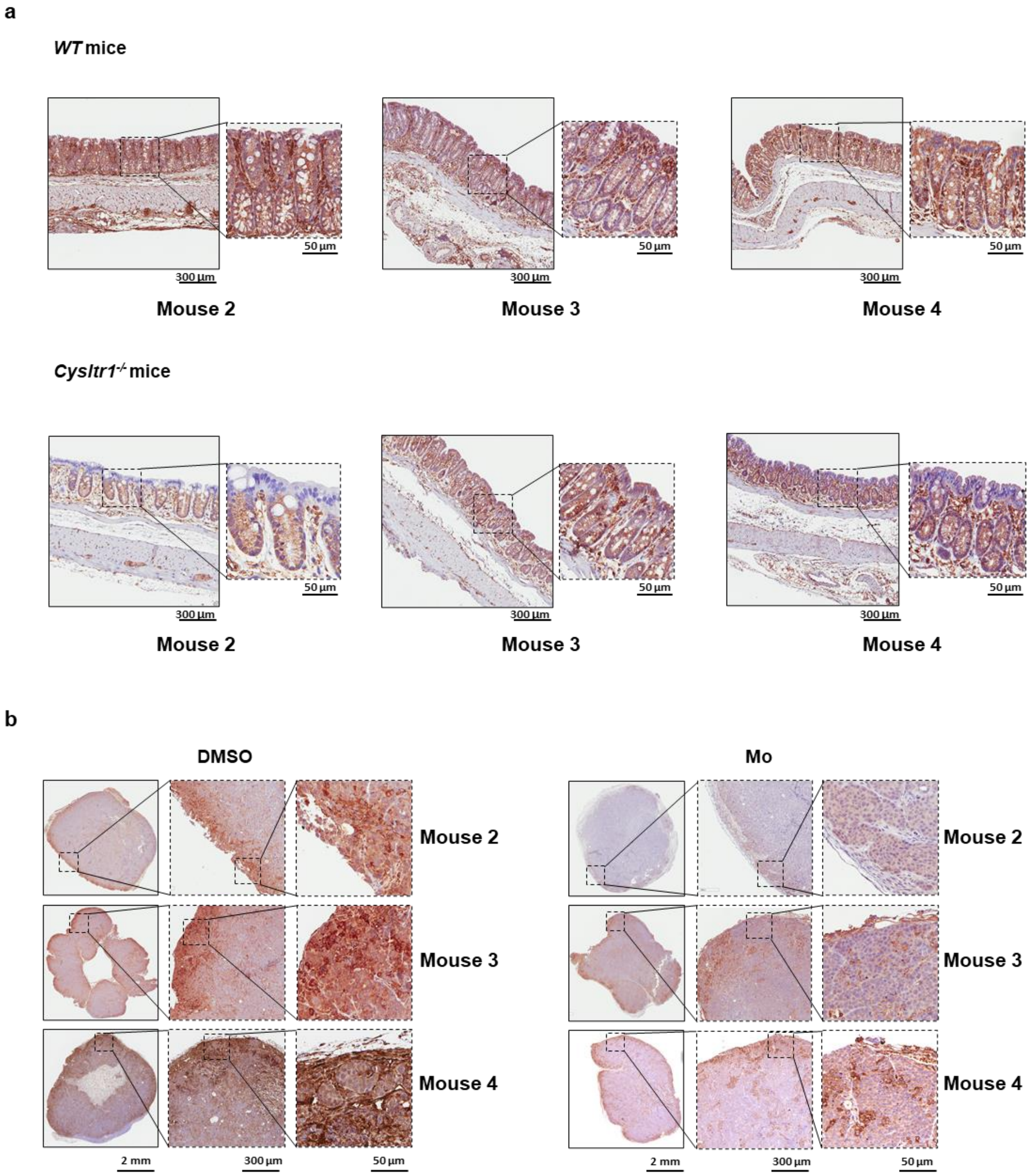

Supplementary Figure 2. Functional absence of CysLT1R negatively regulates BDNF expression. BDNF protein expression from mouse colon tissues of colitis-associated carcinoma (CAC; AOM/DSS-treated) mice. Immunohistochemistry evaluation of colon sections was performed by evaluating six random areas of the whole colon. Representative images (8x and 40x magnification) showing additional 3 mice each for (**a**) wild-type (*WT*) and *Cysltr1* gene disruption (*Cysltr1*$^{-/-}$) group. (**b**) Representative images showing the mouse xenograft section for additional 3 mice each in the group treated with vehicle control (DMSO) or the CysLT1R antagonist, Montelukast (Mo). Scale bars as indicated in the images.

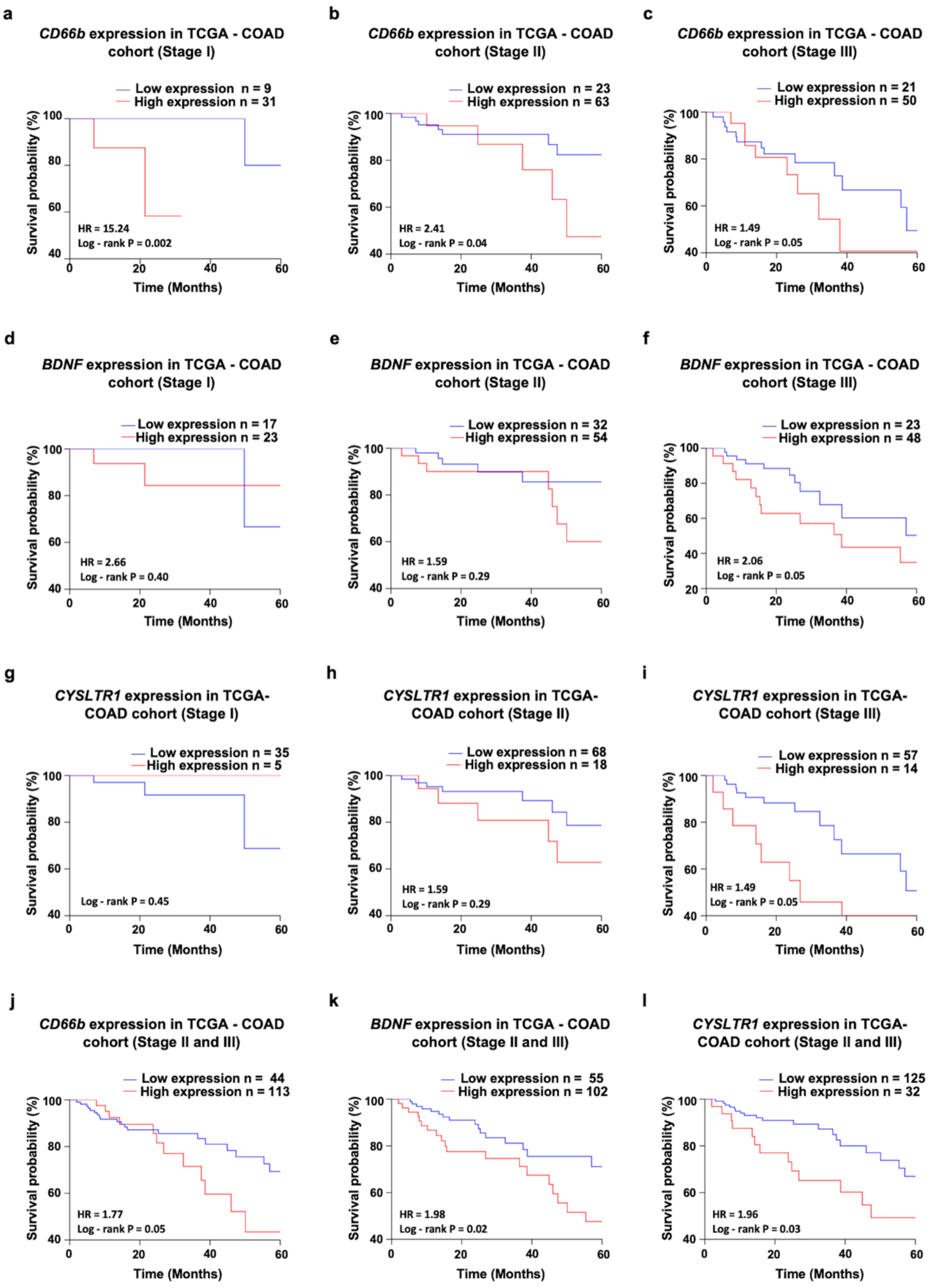

Supplementary Figure 3. Kaplan-Meier survival curves showing gene signature of (**a-c**) *CD66b*, (**d-f**) *BDNF* and (**g-i**) *CYSLTR1* in stage I, II, and III patient groups respectively in TCGA-COAD patient cohort. Survival curves showing gene signature for (**j**) *CD66b*, (**k**) *BDNF* and (**l**) *CYSLTR1* in stage II and III patients group combined in TCGA-COAD patients cohort.

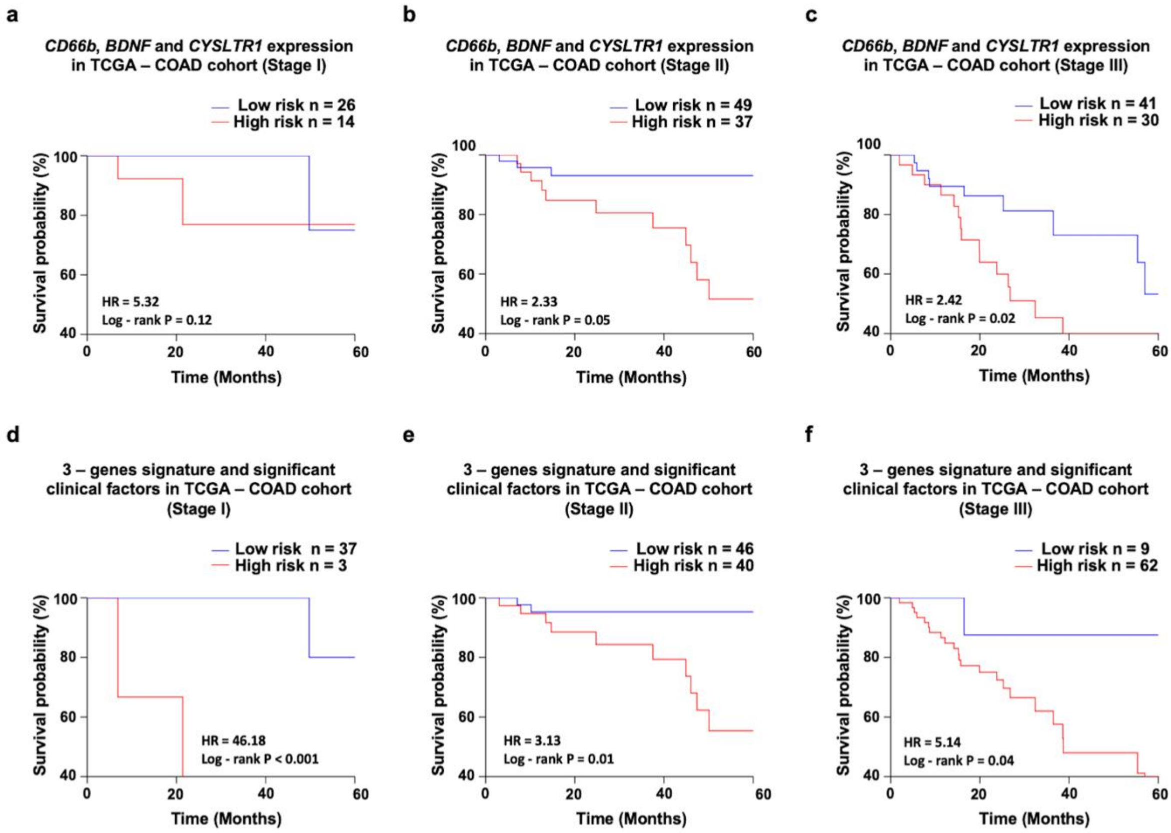

Supplementary Figure 4. Kaplan-Meier survival curves according to (**a-c**) three-gene signature (*CD66b*, *BDNF* and *CYSLTR1*) expression from univariate analysis and (**d-f**) multivariate analysis of the three-gene signature with the significant clinical features (gender, lymph node metastasis and TNM staging) in stage I, II, and I patient groups in TCGA-COAD patient cohort.

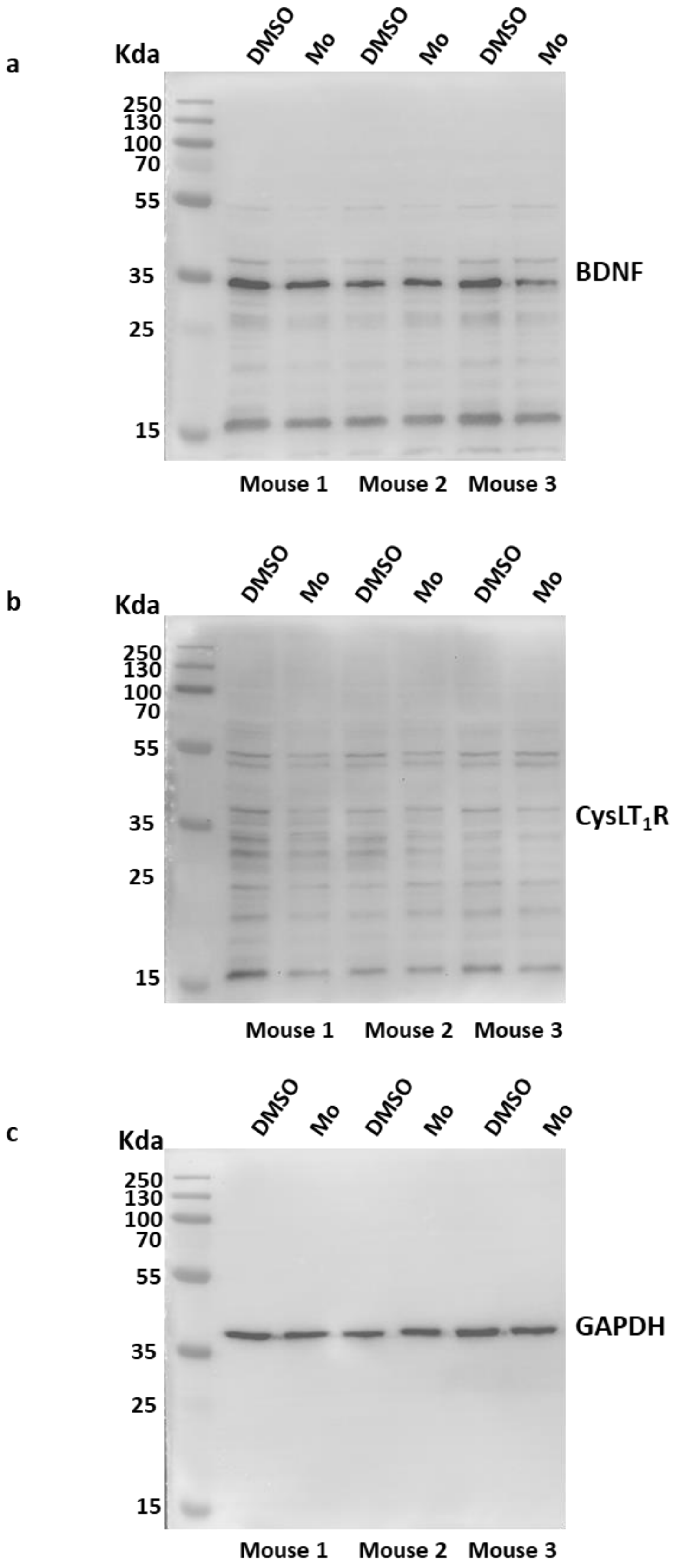

Supplementary Fig. 3. Full Western blot of BDNF (a) $CysLT_1R$ b) and GAPDH (c) protein expression in mice xenograft tumor tissues from DMSO vehicle control or Mo treated mice.
